# Supplementary material for: Porous Polydimethylsiloxane Elastomer Hybrid with Zinc Oxide Nanowire for Wearable, Wide-Range, and Low Detection Limit Capacitive Pressure Sensor
Source: Nanomaterials (Basel). 2022 Jan 14;12(2):256. doi: 10.3390/nano12020256 (PMC8779111; doi:10.3390/nano12020256)
Supplement: Supplementary file 1 [file nanomaterials-12-00256-s001.zip › nanomaterials-1529461-supplementary.pdf]

Article

Porous Polydimethylsiloxane Elastomer Hybrid with Zinc Oxide Nanowire for Wearable, Wide-Range, and Low Detection Limit Capacitive Pressure Sensor

Gen-Wen Hsieh [1,*], Liang-Cheng Shih [2], and Pei-Yuan Chen [2]

1 Institute of Lighting and Energy Photonics, College of Photonics, National Yang Ming Chiao Tung University, 301, Section 2, Gaofa 3rd Road, Guiren District, Tainan 71150, Taiwan
2 Institute of Photonic System, College of Photonics, National Yang Ming Chiao Tung University, 301, Gaofa 3rd Road, Section 2, Guiren District, Tainan 71150, Taiwan; lighttime0625@yahoo.com.tw (L.-C.S.); aazz55255361@gmail.com (P.-Y.C.)
* Correspondence: cwh31@nctu.edu.tw or cwh31@nycu.edu.tw;
Tel.: +86-(0)-6303-2121 (ext. 57797); Fax: +86-(0)-6303-2535

Citation: Hsieh, G.-W.; Shih, L.-C.; Chen, P.-Y. Porous Polydimethylsiloxane Elastomer Hybrid with Zinc Oxide Nanowire for Wearable, Wide-Range, and Low Detection Limit Capacitive Pressure Sensor. *Nanomaterials* **2022**, *12*, 256. https://doi.org/10.3390/nano12020256

Academic Editor: Teresa Cuberes

Received: 13 December 2021
Accepted: 11 January 2022
Published: 14 January 2022

Publisher's Note: MDPI stays neutral with regard to jurisdictional claims in published maps and institutional affiliations.

Copyright: © 2022 by the authors. Licensee MDPI, Basel, Switzerland. This article is an open access article distributed under the terms and conditions of the Creative Commons Attribution (CC BY) license (https://creativecommons.org/licenses/by/4.0/).

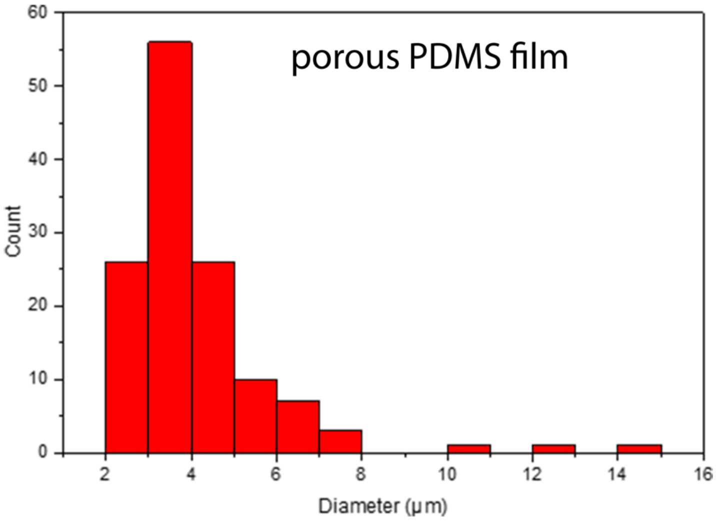

Figure S1. Histograms of diameter distribution of air pores confined in the PDMS (pore size: 4.2 ± 1.8 μm).

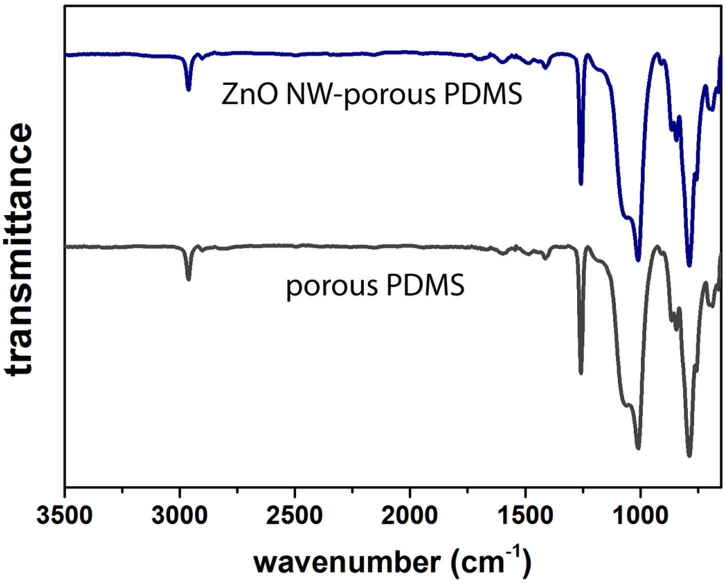

Figure S2. ATR-FTIR spectra of porous PDMS and ZnO nanowire–porous PDMS.

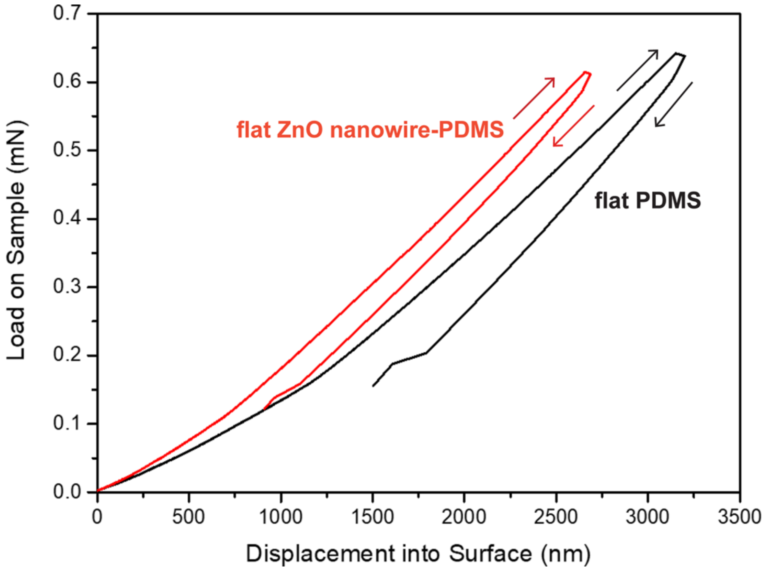

Figure S3. Nanoindentation load-displacement curves for flat PDMS and flat ZnO nanowire–PDMS, respectively (nanoindentation depth: 2 μm; all at a strain rate of 0.05 s^{-1}). Note that we could only acquire the nanoindentation load-displacement curves and elastic modulus of solid flat films because the porous films were too soft to be probed by the Berkovich pyramidal-tip Nanoindenter. The elastic moduli of flat PDMS and flat ZnO nanowire-PDMS were found to be 57 MPa and 68 MPa, respectively; hence, the elastic modulus of PDMS-based composite can be increased by these nanowire shaped fillers. Considerably, we assume that the elastic modulus of ZnO nanowire-porous PDMS nanocomposite could be slightly higher than that of porous PDMS one.

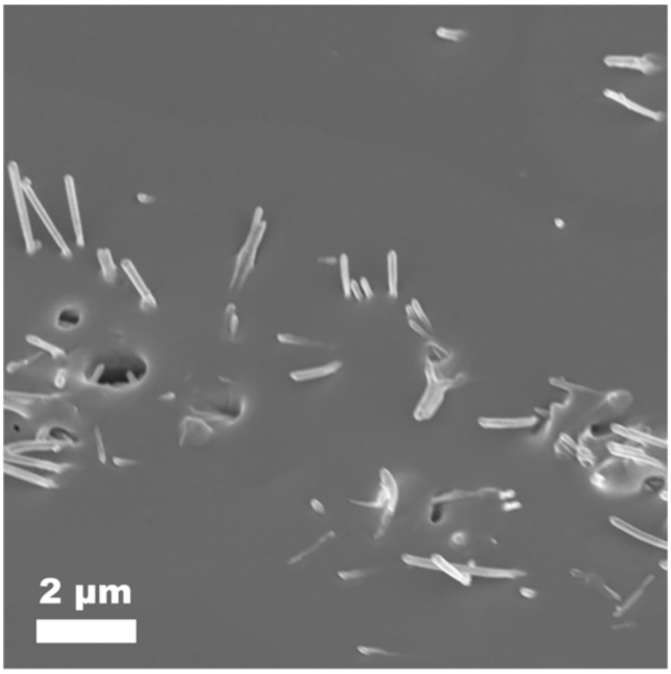

Figure S4. Cross-sectional SEM image of a ZnO nanowire (2 wt%)–porous PDMS film. .

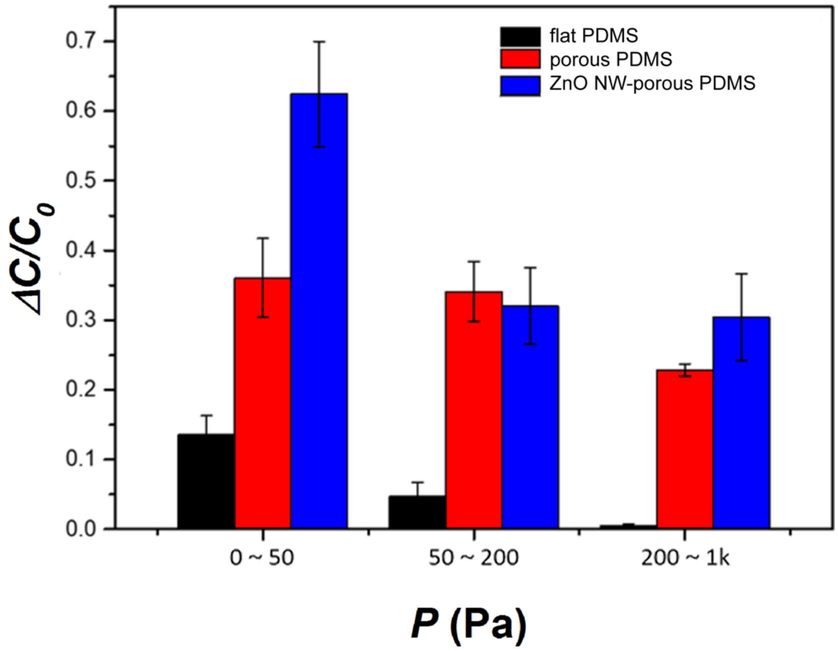

Figure S5. The mean values of relative change in capacitance for flat PDMS, porous PDMS, and ZnO nanowire–porous PDMS capacitive pressure sensors at different pressure regimes, accordingly. .

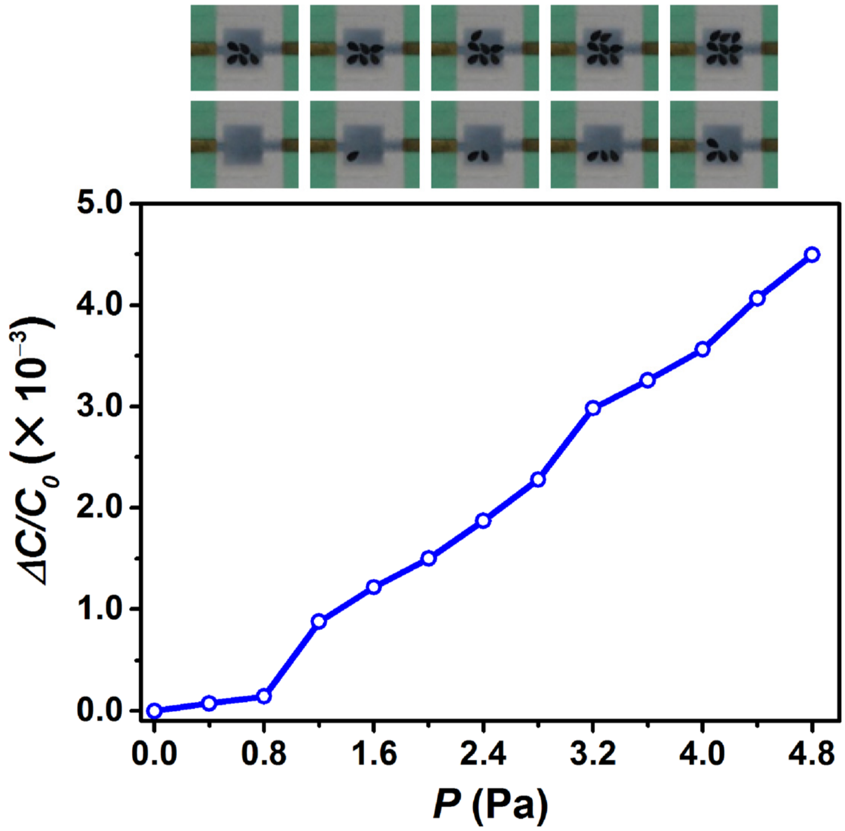

Figure S6. Plots of relative change in capacitance versus applied pressure by means of the sequential placement of sesame seeds (average weight for each seed: ~4 mg). .

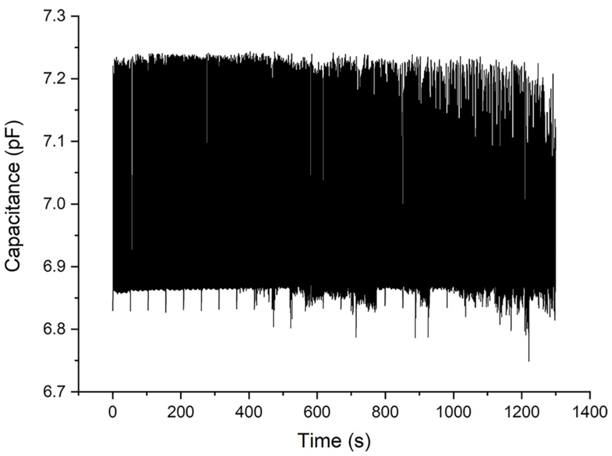

Figure S7. Operational stability of the porous PDMS capacitive pressure sensor with continuous capacitance measurement over 1000 cycles with 300 Pa. The device generally showed stable working stability, but after 600 s (~450 cycles) it revealed slight, unstable capacitance variation. This was probably due to the fatigue-induced degradation occurred to the sensor.

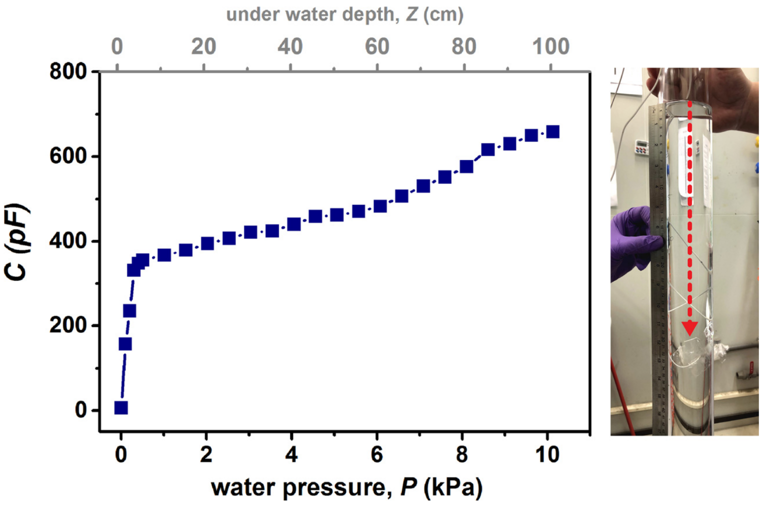

Figure S8. Measured capacitance values as a function of hydrostatic water pressure.

Table S1. Comparison of the ZnO nanowire–porous PDMS device with other recently reported capacitive pressure sensors.

dielectric	electrode/substrate	max sensitivity (kPa^{-1})	limit of detection (Pa)	response time (ms)	operation range	ref.
PDMS	Ag NW/PET	0.0004 (<50 kPa)	5	2000	50 Pa–260 kPa	[1]
Pyramidal PDMS	ITO/PET	0.55 (<2 kPa)	3	<<1 s	30 Pa–20 kPa	[2]
PDMS/Ecoflex	CNT/PDMS	0.23	50	125	50 Pa–900 kPa	[3]
Ag-PDMS	Ag	0.00145	6	–	6 Pa–100 kPa	[4]
Ag NP–porous PDMS	ITO/PET	0.11	8	28	8 Pa–100 kPa	[5]
graphene–PDMS sponge	Cu/PI	0.12 (<10 kPa)	5	7	5 Pa–500 kPa	[6]
micropillar PDMS	Au/PET	0.42 (<1.5 kPa)	1	70	1 Pa–13 kPa	[7]
parylene/PU nanomesh	Au nanomesh	0.141 (< 1 kPa)	–	190	Up to 100 kPa	[8]
porous PDMS + air gap	SWNT/PDMS	1.5 (<1 kPa)	2.5	1000	2.5 Pa–20 kPa	[9]
Porous Ecoflex	CNT/Ecoflex	0.601 (<5 kPa)	0.1–0.2	–	0.1 Pa–130 kPa	[10]
carbon black–porous PDMS/PDMS	CNT/PDMS	35 (< 200 Pa)	9	–	1 Pa–12 kPa	[11]
porous pyramid PDMS	ITO/PET	44.5 (< 100 Pa)	0.14	50	0.1 Pa–130 kPa	[12]
ZnO NW–porous PDMS	PEDOT:PSS/PET	0.717 (<50 Pa)	1.0 Pa with 0.4 Pa resolution	260	0.4 Pa–50 kPa	**this work**

References

1. B. Zhang, Z. Xiang, S. Zhu, Q. Hu, Y. Cao, J. Zhong, Q. Zhong, B. Wang, Y. Fang, and B. Hu, Dual functional transparent film for proximity and pressure sensing, *Nano Research*, 2014, **7**, 1488–1496.
2. S.C.B. Mannsfeld, B.C.-K. Tee, R.M. Stoltenberg, C.V.H.-H. Chen, S. Barman, B.V.O. Muir, A.N. Sokolov, C. Reese and Z. Bao, Highly sensitive flexible pressure sensors with microstructured rubber dielectric layers, *Nat. Mater.*, 2010, **9**, 859–864.
3. D. Lipomi, M. Vosgueritchian, B.C.-K. Tee, S.L. Hellstrom, J.A. Lee, C.-H. Fox and Z. Bao, Skin-like pressure and strain sensors based on transparent elastic films of carbon nanotubes, *Nat. Nanotechnol.*, 2011, **5**, 788–792.
4. X. Zhao, Q. Hua, R. Yu, Y. Zhang, and C. Pan, Flexible, stretchable and wearable multifunctional sensor array as artificial electronic skin for static and dynamic strain mapping, *Adv. Electron. Mater.*, 2015, **1**, 1500142.
5. S.-Y. Liu, J.-G. Lu, and H.-P. D. Shieh, Influence of permittivity on the sensitivity of porous elastomer-based capacitive pressure sensors, *IEEE Sensors J.*, 2018, **18**, 1870–1876.
6. H. Kou, L. Zhang, Q. Tan, G. Liu, H. Dong, W. Zhang and J. Xiong, Wireless wide-range pressure sensor based on graphene/PDMS sponge for tactile monitoring, *Scientific Reports*, 2019, **9**, 3916.
7. Y. Luo, J. Shao, S. Chen, C. Chen, H. Tian, X. Li, L. Wang, D. Wang and B. Lu, Flexible capacitive pressure sensor enhanced by tilted micropillar arrays, *ACS Appl. Mater. Interfaces*, 2019, **11**, 17796–17803.
8. D. Kwon, T.-I. Lee, J. Shim, S. Ryu, M.S. Kim, S. Kim, T.-S. Kim and I. Park, Highly sensitive, flexible, and wearable pressure sensor based on a giant piezocapacitive effect of three-dimensional microporous elastomeric layer, *ACS Appl. Mater. Interfaces*, 2016, **8**, 16922–16931.
9. S. Lee, S. Franklin, F.A. Hassani, T. Yokota, M.O.G. Nayeem, Y. Wang, R. Leib, G. Cheng, D.W. Franklin, T. Someya, Nanomesh pressure sensor for monitoring finger manipulation without sensory interference, *Science*, 2020, **370**, 966–970.
10. S. Park, H. Kim, M. Vosgueritchian, S. Cheon, H. Kim, J. H. Koo, T. R. Kim, S. Lee, G. Schwartz, H. Chang and Z. Bao, Stretchable energy-harvesting tactile electronic skin capable of differentiating multiple mechanical stimuli modes, *Adv. Mater.*, 2014, **26**, 7324–7332.
11. M. Pruvost, W.J. Smit, C. Monteux, P. Poulin and A. Colin, Polymeric foams for flexible and highly sensitive low-pressure capacitive sensors, *npj Flexible Electronics*, 2019, **3**, 7.
12. J.C. Yang, J.-O. Kim, J. Oh, S. Y. Kwon, J.Y. Sim, D.W. Kim, H.B. Choi and S. Park, Microstructured porous pyramid-based ultrahigh sensitive pressure sensor insensitive to strain and temperature, *ACS Appl. Mater. Interfaces*, 2019, **11**, 19472–19480.
